# Prognostic role of long noncoding RNA ZFAS1 in cancer patients: a systematic review and meta-analysis

**DOI:** 10.18632/oncotarget.19162

**Published:** 2017-07-11

**Authors:** Tian Lan, Xiong Lan, Guangcai Li, Zhen Zheng, Minghua Zhang, Faxiang Qin

**Affiliations:** ^1^ Department of Hepatobiliary Surgery, Zhongnan Hospital of Wuhan University, Wuhan, Hubei, 430071, P.R. China; ^2^ Department of Respiratory Medicine, The Central Hospital of Enshi Tujia and Miao Autonomous Prefecture, Enshi, Hubei, 445000, P.R. China

**Keywords:** lncRNA, ZFAS1, cancer, prognosis, meta-analysis

## Abstract

Long noncoding RNA ZFAS1 has been identified as a crucial role in the tumorigenesis of malignant tumors. Numerous studies reported that the expression levels of ZFAS1 in tumor tissues were dramatically higher than that in adjacent normal tissues. We conducted a meta-analysis to investigate the correlation between ZFAS1 expression and clinical outcomes of cancer patients. The databases of PubMed, EMBASE, Web of Science, Cochrane Library, CNKI and WanFang were retrieved for eligible studies. A total of 841 patients from 9 studies were eventually included. Our results demonstrated that increased ZFAS1 expression was significantly associated with poor OS in cancer patients (HR = 2.13, 95% CI = 1.71–2.65, *P* < 0.001). Patients with high ZFAS1 expression presented shorter RFS than those with low ZFAS1 expression (HR = 2.00, 95% CI = 1.45–2.77, *P* < 0.001). The clinicopathological parameters analysis demonstrated that increased ZFAS1 expression was significantly associated with vascular invasion (OR = 2.26, 95% CI = 1.36–3.78, *P* = 0.002), lymph node metastasis (OR = 2.98, 95% CI = 2.12–4.19, *P* < 0.001) and advanced TNM stage (OR = 3.00, 95% CI = 2.18–4.12, *P* < 0.001). In conclusion, lncRNA ZFAS1 might serve as a prognostic biomarker for cancer patients and increased ZFAS1 expression may be closely related to advanced characteristics of cancer.

## INTRODUCTION

As one of the most serious diseases wreaking havoc on human health, cancer has now served as the leading cause of morbidity and mortality in most areas worldwide [1]. Since early diagnosis greatly affect the survival rate of cancer patients and the prognosis of various cancers remains very poor, accumulating researchers are attempting to identify the key biological factors involved in the development and progression of this fatal disease [2]. Nevertheless, few molecular targets have been deemed as biomarkers for diagnosis and prognosis in clinical application. Therefore, identifying reliably diagnostic and prognostic markers for cancer is urgently needed. Nowadays, an increasing number of studies reported the role of non-coding RNAs (ncRNAs) in cancer, including long noncoding RNA (lncRNAs), micro-RNAs (miRNAs) and small nucleolar RNAs (snoRNAs) [3–5].

Long noncoding RNAs (lncRNAs) are a class of RNA molecule with more than 200 nucleotides in length and do not code for proteins but bear the ability to increase and/or decrease the gene expressions^3^. Accumulating evidence showed that lncRNAs have been identified to activate and/or inhibit multiple biological regulatory processes, including development, differentiation and carcinogenesis [6, 7]. As a newly identified lncRNA, zinc finger antisense 1 (ZFAS1) has been found in different cancers, including breast cancer, colorectal cancer (CRC), gastric cancer (GC) and hepatocellular carcinoma (HCC) [8, 9]. More importantly, the dysregulation of ZFAS1 was closely related to cell cycle control and apoptosis of cancer cells [10]. However, most studies investigating the implications of ZFAS1 expression are limited by small sample size. In addition, no systematic review or meta-analysis has been performed to assess the association between ZFAS1 expression and the prognosis of patients with cancers. Thus, we conducted a systematic review and meta-analysis to explore the prognostic role of ZFAS1 in cancers patients.

## MATERIALS AND METHODS

### Literature search strategies

An electronic search was performed in PubMed, EMBASE, Web of Science, Cochrane Library as well as Chinese databases including CNKI and WanFang database for all the relevant studies which reported the association between lncRNA ZFAS1 expression and clinical outcomes in different cancers by utilizing the following search strings: “long non-coding RNA ZFAS1” or “lncRNA ZFAS1” or “ZFAS1” or “Zinc finger antisense 1”, “cancer or carcinoma or tumor or neoplasm”, and “pathology”. These terms were used in different combinations. No limitation was placed on publication status or language.

### Inclusion and exclusion criteria

The eligible studies met the following criteria: (1) any kind of human cancer was studied; (2) the study provided at least one of following clinical outcomes: gender, tumor size, vascular invasion (VI), lymph node metastasis (LNM), TNM stage, recurrence-free survival (RFS) and overall survival (OS); (3) the method of detecting lncRNA ZFAS1 was restricted to quantitative real-time reverse transcription polymerase chain reaction (qRT-PCR); (4) patients were divided into high and low groups based on the expression levels of ZFAS1. Exclusion criteria were as follows: (1) reviews, case reports, meta-analysis, and duplicate publications; (2) the studies without usable data; (3) the studies only focusing on the molecular mechanism of lncRNA ZFAS1.

### Data extraction and quality assessment

Two investigators independently extracted the data from each original publication including first author’s name, year of publication, country of origin, cancer type, total cases, numbers of patients in high and low ZFAS1 expression groups, the detection method, outcome measures and the cut-off value for ZFAS1 levels. Missing information was estimated according to the Cochrane Handbook and was requested from the authors of original studies if necessary. Discrepancies between the 2 investigators were resolved by discussion and consensus. The quality of included studies was assessed via using Newcastle-Ottawa Scale (NOS) standard [11], which includes selection (4 points), comparability (2 points) and outcome (3 points) with a score range of 0∼9. A study with NOS score more than 6 was considered to be of high quality.

### Statistical analysis

Odds ratios (ORs) with 95% confidence intervals (CIs) were estimated to evaluate the correlation between ZFAS1 expression and the clinical outcomes in cancer patients. According to the American Joint Committee on Cancer (AJCC) staging system [12], TNM stage was separated into two groups, which were early stage (I–II) and advanced stage (III–IV). Additionally, lymph node metastasis (LNM) was divided into “positive” and “negative” groups. Statistical analysis was performed by utilizing RevMan 5.3 software and Stata SE12.0 (Stata Corporation). Cochrane *Q*-test and *P* values were used to determine the heterogeneity across studies. If heterogeneity was present (*I*^2^ ≥ 50% or *P* < 0.05), random-effect model was used to pool the results. Conversely, the fixed-effect model was applied for the analysis [13]. As for the acquisition of hazard ratios (HRs) of survival, the data were directly extracted from original articles. If not applicable, the data were calculated by using Engauge Digitizer version 4.1 (http://digitizer.sourceforge.net/) from Kaplan-Meier curves [14]. The publication bias was evaluated utilizing funnel plot, and *P* < 0.05 was considered as the existence of publication bias.

## RESULTS

### Characteristics of included studies

**Figure 1:**
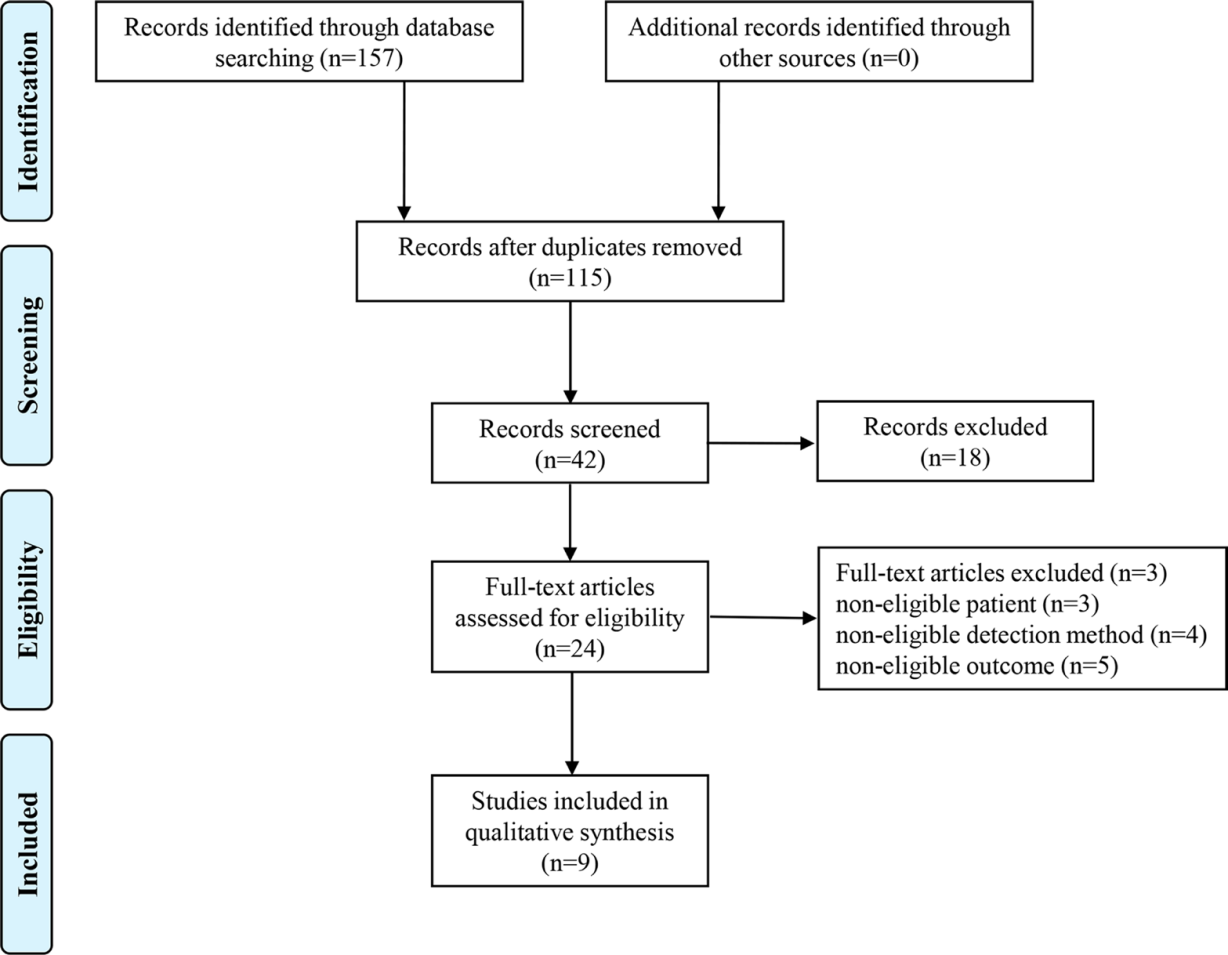
Flow chart of the study search and selection

As shown in Figure 1, 157 articles were found according to the search strategy and then 115 duplicates are removed. Subsequently, 18 records without full-text were excluded. After reading the abstracts and full-texts of the remaining 24 articles, 15 records not conforming to the inclusion criteria were further excluded. Eventually, 9 studies [15–23] involving a total of 841 patients met the inclusion criteria. All included studies came from China, in which six different types of cancers were detected, with two cases of gastric cancer, two cases of colorectal cancer, one case of hepatocellular carcinoma, two cases of glioma, one case of non-small cell lung cancer and one case of epithelial ovarian cancer. The expression level of ZFAS1 was determined in collected tumor tissues and adjacent normal tissues by using qRT-PCR. The cut-off values for ZFAS1 expression included median and fold change. Each study was evaluated to be of high quality (≧ 6). The characteristics of the included studies were presented in Table 1.

**Table 1:** Characteristics of studies included in this meta-analysis

First author	Year	Country	Cancer type	Total cases	ZFAS1 expression		Detection method	Outcome measures	Cut-off (high/low)	Quality score
					High	Low				
Li T	2015	China	HCC	113	57	56	qRT-PCR	OS, RFS	median	8
Fang C	2016	China	CRC	73	36	37	qRT-PCR	N/A	median	7
Nie F	2016	China	GC	54	27	27	qRT-PCR	OS, RFS	median	8
Tian FM	2016	China	NSCLC	173	85	88	qRT-PCR	OS	N/A	7
Wang W	2016	China	CRC	159	79	80	qRT-PCR	OS, RFS	median	6
Gao K	2017	China	glioma	46	23	23	qRT-PCR	OS	median	6
Lv QL	2017	China	glioma	69	27	42	qRT-PCR	OS	N/A	8
Pan L	2017	China	GC	94	58	36	qRT-PCR	N/A	fold ≧2.0	6
Xia B	2017	China	EOC	60	30	30	qRT-PCR	OS	median	8

HCC: hepatocellular carcinoma; CRC: colorectal cancer; GC: gastric cancer; NSCLC: non-small cell lung cancer; EOC: epithelial ovarian cancer; qRT-PCR: quantitative real-time reverse transcription polymerase chain reaction; OS: overall survival; RFS: recurrence-free survival.

### Association between ZFAS1 expression and overall survival (OS)

**Figure 2:**
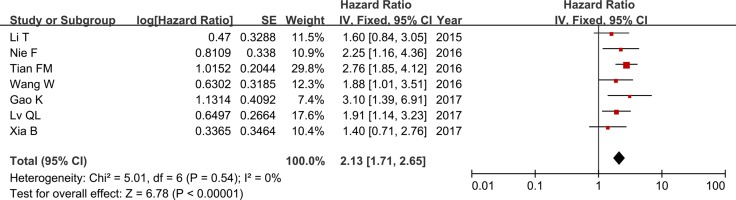
Forest plot of HRs for the association between ZFAS1 expression and OS in cancer patients

A total of seven studies reported the correlation between ZFAS1 expression and OS. Because of no statistical heterogeneity (*P* = 0.54, *I*^2^ = 0.0%), the fixed-effects model was chosen to estimate the pooled HRs and 95% CIs. The HR of the high ZFAS1 expression group versus low ZFAS1 expression group was 2.13 (95% CI = 1.71–2.65, *P* < 0.001, Figure 2). In other words, compared with low ZFAS1 expression group, high ZFAS1 expression group presented a statistically significant shorter OS, which implied that increased ZFAS1 expression was significantly associated with poor OS.

**Figure 3:**
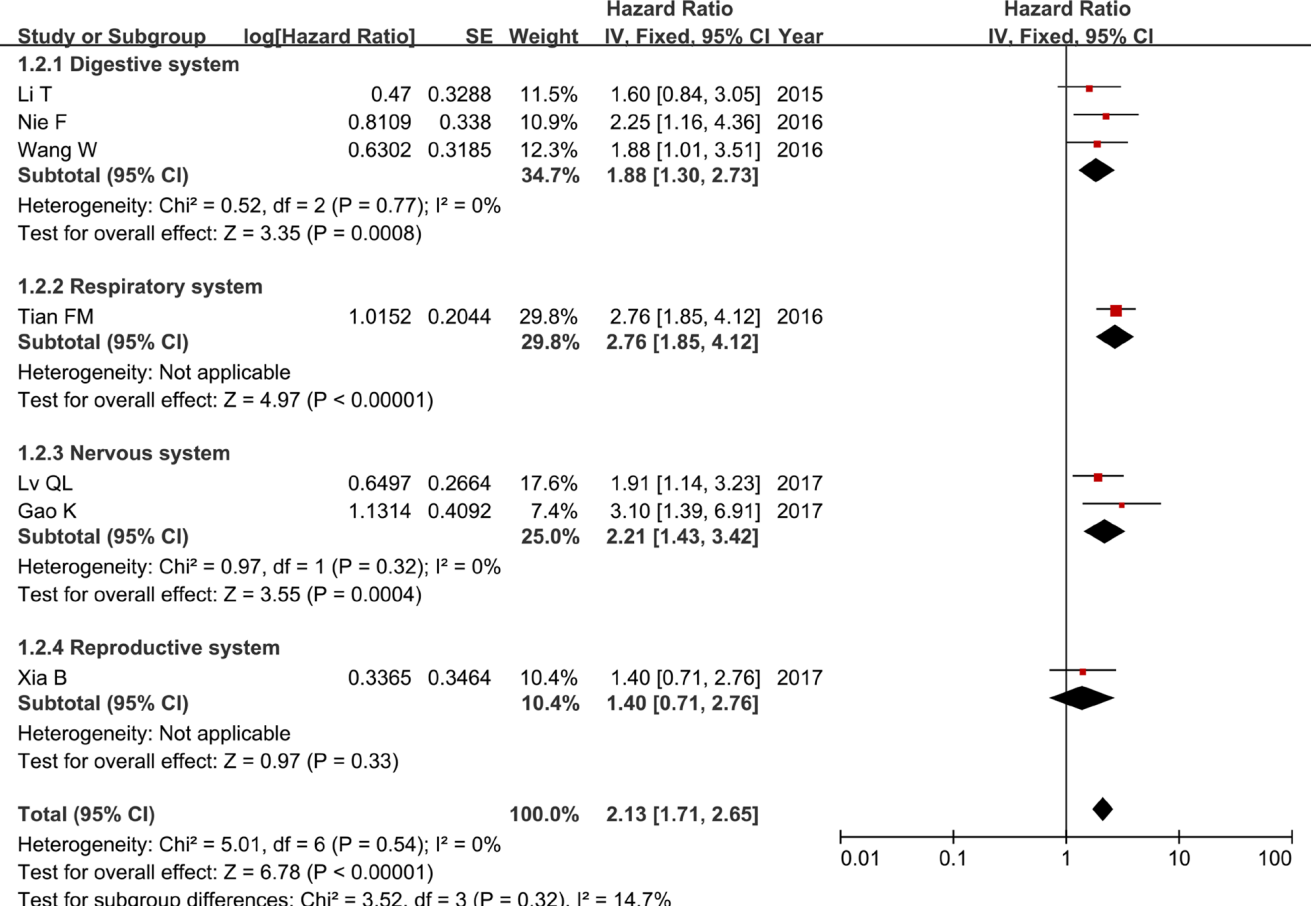
Forest plot of HRs for the association between ZFAS1 expression and OS in subgroup analysis based on different cancer types

As shown in Table 1, six different types of cancer (gastric cancer, colorectal cancer, hepatocellular carcinoma, glioma, non-small cell lung cancer and epithelial ovarian cancer) were included in this meta-analysis. Hence, we classified these studies into four subgroups (digestive system, nervous system, respiratory system and reproductive system) based on cancer types. As no significant heterogeneity among studies in different subgroups was detected, we used fixed-effects model to complete the synthesis of data. The results of subgroup analysis demonstrated that the HRs of the high ZFAS1 expression group versus the low ZFAS1 expression group in digestive system cancers, nervous system cancers, respiratory system cancers and reproductive system cancers were 1.88 (95% CI = 1.30–2.73, *P* < 0.001), 2.21 (95% CI = 1.43–3.42, *P* < 0.001), 2.76 (95% CI = 1.85–4.12, *P* < 0.001) and 1.40 (95% CI = 0.71–2.76, *P* = 0.33), respectively (Table 2 and Figure 3). Our data suggested that increased ZFAS1 expression in digestive system cancers, nervous system cancers and respiratory system cancers was statistically associated with shorter OS, whereas there was no significant difference of OS between high ZFAS1 expression group and low ZFAS1 expression group in reproductive system cancers (Table 2 and Figure 3).

**Table 2:** Subgroup meta-analysis of pooled HRs for OS

Categories	Studies (*n*)	Total cases	Fixed-effects model		Heterogeneity	
			HR (95% CI) for OS	*P*-value	*I*^2^ (%)	*P*_h_
[1] OS	7	674	2.13 (1.71–2.65)	< 0.001	0	0.54
[2] Cancer type						
1) Digestive system	3	326	1.88 (1.30–2.73)	< 0.001	0	0.77
2) Nervous system	2	115	2.21 (1.43–3.42)	< 0.001	0	0.32
3) Respiratory system	1	173	2.76 (1.85–4.12)	< 0.001	N/A	N/A
4) Reproductive system	1	60	1.40 (0.71–2.76)	0.33	N/A	N/A
[3] Cut-off (high/low)						
Median	5	432	1.91 (1.41–2.58)	< 0.001	0	0.60
Others	2	242	2.41 (1.75–3.31)	< 0.001	16	0.28
[4] Sample sizes						
> 100	3	445	2.25 (1.67–3.03)	< 0.001	17	0.30
≤ 100	4	229	2.00 (1.45–2.76)	< 0.001	0	0.50
[5] Duration of follow-up						
≤ 40 months	2	167	1.89 (1.19–3.00)	0.007	0	0.47
> 40 months	5	507	2.21 (1.72–2.83)	< 0.001	4	0.39

**Figure 4:**
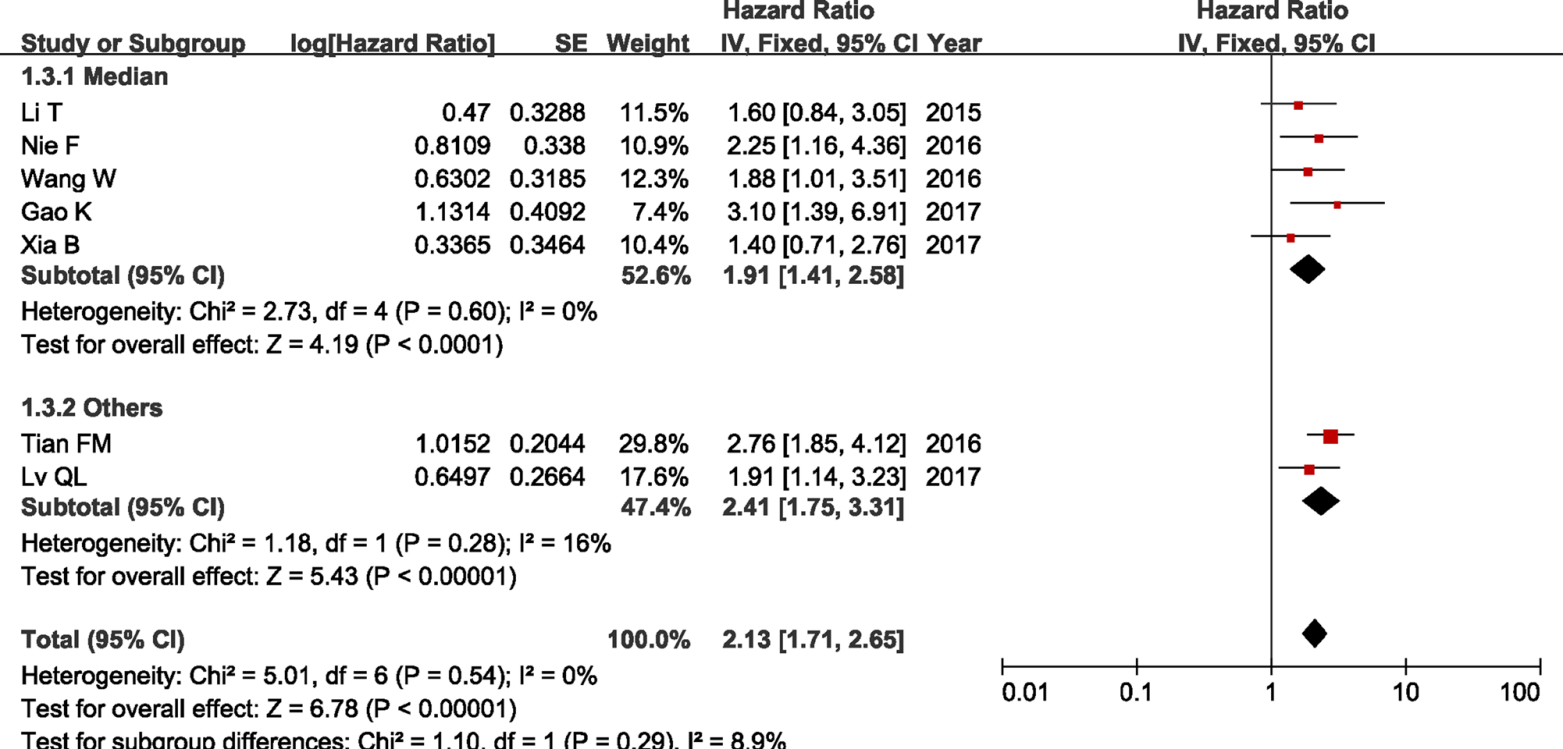
Forest plot of HRs for the association between ZFAS1 expression and OS in subgroup analysis based on cut-off (high/low)

**Figure 5:**
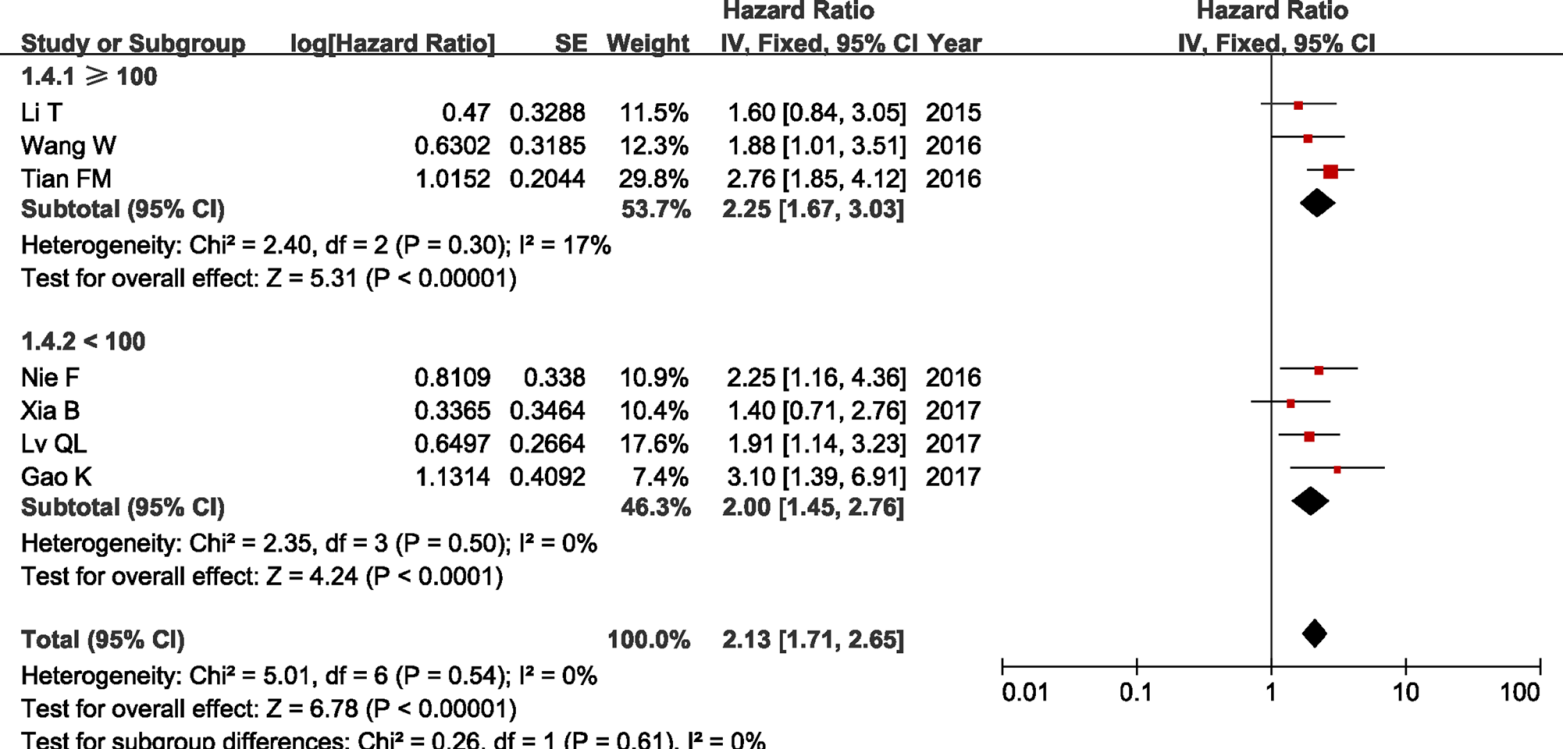
Forest plot of HRs for the association between ZFAS1 expression and OS in subgroup analysis based on sample sizes

**Figure 6:**
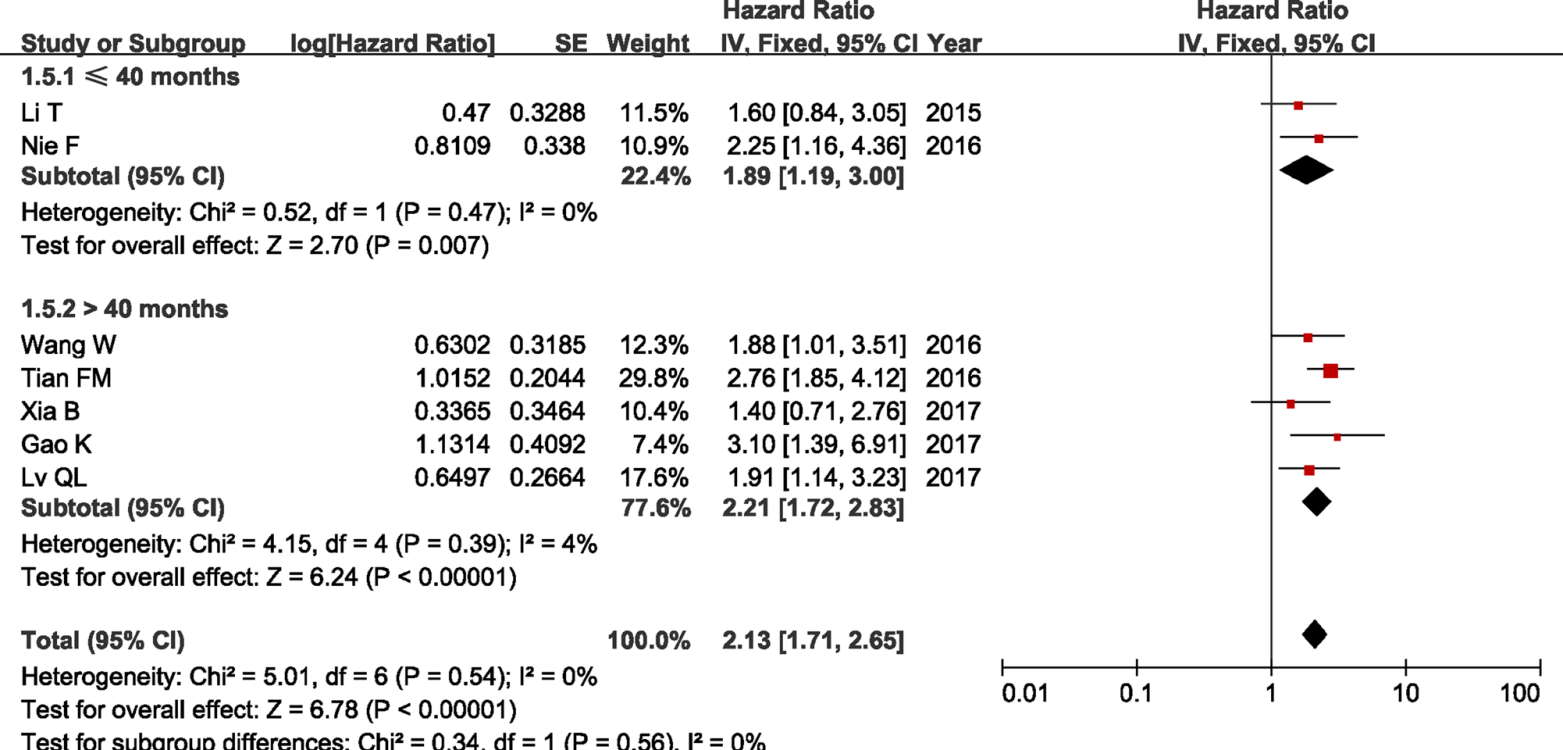
Forest plot of HRs for the association between ZFAS1 expression and OS in subgroup analysis based on duration of follow-up

In addition, we conducted a series of subgroup meta-analysis based on cut-off (high/low), sample sizes and duration of follow-up. Since the heterogeneity among studies in all subgroups did not present statistically significant, the fixed-effects model was adopted to calculate the pooled HR and 95% CI. The results revealed that increased ZFAS1 expression was significantly associated with poor OS in all subgroup meta-analysis (Table 2 and Figures 4, 5 and 6).

### Association between ZFAS1 expression and recurrence-free survival (RFS)

**Figure 7:**
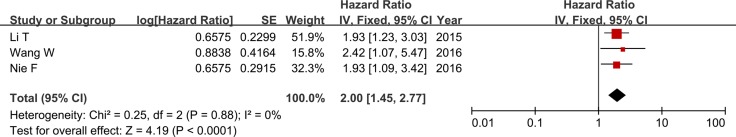
Forest plot of HRs for the association between ZFAS1 expression and RFS in cancer patients

A total of three studies described the association between ZFAS1 expression and RFS. Due to no statistically significant heterogeneity (*P* = 0.88, *I*^2^ = 0.0%), the fixed-effects model was adopted to calculate the pooled HR with corresponding 95% CI. Our results indicated that RFS was significantly shorter in high ZFAS1 expression group compared with that in low ZFAS1 expression group (HR = 2.00, 95% CI = 1.45–2.77, *P* < 0.001, Figure 7).

### Association between ZFAS1 expression and clinicopathological parameters

We collected all the clinicopathological data from these eligible studies to perform further meta-analysis for the correlation between ZFAS1 expression and clinicopathological parameters. As presented in Table 3, increased ZFAS1 expression was significantly associated with vascular invasion (OR = 2.26, 95% CI = 1.36–3.78, *P* = 0.002, Supplementary Figure 1), lymph node metastasis (OR = 2.98, 95% CI = 2.12–4.19, *P* < 0.001, Supplementary Figure 2) and advanced TNM stage (OR = 3.00, 95% CI = 2.18–4.12, *P* < 0.001, Supplementary Figure 3). Conversely, no statistical correlation was observed between ZFAS1 expression with gender (*P* = 0.91, Supplementary Figure 4) and tumor size (*P* = 0.05, Supplementary Figure 5). Due to insufficient data for other clinicopathological parameters (such as age, distant metastasis, tumor differentiation), the association between ZFAS1 expression and these clinicopathological parameters were not evaluated for the meta-analysis.

**Table 3:** Meta-analysis of the association between ZFAS1 expression and clinicopathological parameters

Clinicopathological parameters	Studies (*n*)	Total cases	OR (95% CI)	*P*-value	Heterogeneity		
					*I*^2^ (%)	*P*_h_	Model
Gender (Male vs. Female)	7	701	0.98 (0.73–1.33)	0.91	15	0.32	Fixed
Tumor size (≥ 5 cm vs. < 5 cm)	5	539	1.41 (1.00–1.99)	0.05	24	0.26	Fixed
Vascular invasion (Yes vs. No)	3	280	2.26 (1.36–3.78)	0.002	0	0.52	Fixed
Lymph node metastasis (Yes vs. No)	6	613	2.98 (2.12–4.19)	< 0.001	11	0.35	Fixed
TNM stage ( III–IV vs. I–II)	7	679	3.00 (2.18–4.12)	< 0.001	2	0.41	Fixed

### Publication bias and sensitivity analysis

Funnel plot analysis was performed to evaluate publication bias for the association between ZFAS1 expression and OS in cancer patients.in the study. The results showed that no obvious publication bias was observed in the included studies for this meta-analysis (Supplementary Figure 6). Sensitivity analysis was conducted to determine whether the individual study exerted influence on the overall results. Our data suggested that removing any of the included studies had no significant influence on the results (Supplementary Figure 7), which demonstrated that our results were considerably reasonable and reliable.

## DISCUSSION

Previous studies reported that lncRNAs played crucial roles in the process of gene expression and their dysregulation were involved in the tumorigenesis and progression of malignant tumors [24, 25]. Notably, antisense lncRNAs (aslncRNAs), oriented in antisense direction with respect to a protein coding loci, also serve as key regulators of genes located on the opposite strand [26]. Growing number of studies identified that aslncRNAs functioned as promoters or enhancers of cancer-related gene expressions in the complex network of signaling pathways in different cancer cell [27, 28].

LncRNA zinc finger antisense 1 (ZFAS1) locus is host to three C/D-box small nucleolar RNAs (snoRNAs), and its transcription starts from the antisense strand near the 5′end of the protein-coding gene Znfx1 [29]. Numerous studies reported that the expression levels of ZFAS1 in tumor tissues were dramatically higher than that in adjacent normal tissues [16, 18]. Additionally, the depletion of ZFAS1 could substantially suppress the proliferation, invasion and migration of cancer cells, while the overexpression of ZFAS1 could markedly promote the tumorigenesis and metastasis [19]. As for the specific molecular mechanism, Li, T et al. found that ZFAS1 increased ZEB1, MMP14 and MMP16 expression and promoted HCC metastasis by sponging miR-150 and inhibiting its function [23]. Furthermore, silenced ZFAS1 could impair migration and invasion by inhibiting the epithelial–mesenchymal transition through reducing the expression of MMP2, MMP9, N-cadherin, Integrin β1, ZEB1, Twist, and Snail as well as increasing E-cadherin level in glioma [21]. Besides, Thorenoor, N et al. identified ZFAS1 as an oncogene via destabilization of p53 and through interaction with CDK1/cyclin B1 complex leading to cell cycle progression and inhibition of cell apoptosis in CRC cells [10].

This meta-analysis was to investigate the prognostic role of lncRNA ZFAS1 in cancer patients. Based on the analysis results, we found that increased ZFAS1 expression was significantly associated with poor OS in cancer patients. Further subgroup meta-analysis demonstrated that ZFAS1 could be a reliable prognostic biomarker for digestive system cancers, nervous system cancers and respiratory system cancers. Likewise, patients with high ZFAS1 expression presented shorter RFS than those with low ZFAS1 expression. Besides, we conducted the meta-analysis for the association between ZFAS1 expression and clinicopathological parameters. The results showed that patients with high ZFAS1 expression had a higher risk of vascular invasion and they suffered higher possibility of developing lymph node metastasis and advanced TNM stage (III–IV), indicating that increased ZFAS1 expression may be closely related to advanced characteristics of cancer.

However, there were several limitations that should be considered in our analysis. To begin with, all studies came from China, which made the included cases could not represent all cancer patients to some extent. Next, the cut-off value for dividing patients into high and low ZFAS1 expression groups was not consistent across studies. Finally, no prospective study was included for further investigation. Therefore, large-scale, multicenter and high-quality studies are supposed to confirm our analysis results in the future.

In conclusion, this meta-analysis suggested that lncRNA ZFAS1 might serve as a prognostic biomarker for cancer patients. Furthermore, increased ZFAS1 expression may be closely related to advanced characteristics of cancer. Nonetheless, due to the limitations in our analysis, large-scale, multicenter and high-quality studies are required to support our results in the future.

## SUPPLEMENTARY MATERIALS FIGURES


