# Supplementary material for: Prognostic role of long noncoding RNA ZFAS1 in cancer patients: a systematic review and meta-analysis
Source: Oncotarget. 2017 Jul 11;8(59):100490–8. doi: 10.18632/oncotarget.19162 (PMC5725037; doi:10.18632/oncotarget.19162)
Supplement: Supplementary file 1 [file oncotarget-08-100490-s001.pdf]

Prognostic role of long noncoding RNA ZFAS1 in cancer patients: a systematic review and meta-analysis

SUPPLEMENTARY MATERIALS

	High ZFAS1 expression		Low ZFAS1 expression			Odds Ratio	
Study or Subgroup	Events	Total	Events	Total	Weight	M-H, Fixed, 95% CI	Year
Li T	29	57	20	56	49.9%	1.86 [0.88, 3.96]	2015
Fang C	17	36	7	37	18.4%	3.83 [1.34, 10.97]	2016
Pan L	21	58	8	36	31.7%	1.99 [0.77, 5.14]	2017
**Total (95% CI)**		**151**		**129**	**100.0%**	**2.26 [1.36, 3.78]**	
Total events	67		35				

Heterogeneity: Chi² = 1.29, df = 2 (P = 0.52); I² = 0%
Test for overall effect: Z = 3.13 (P = 0.002)

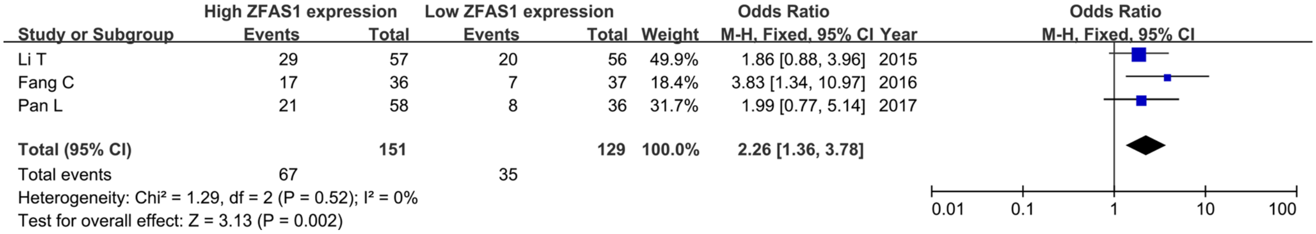

Supplementary Figure 1: Forest plot of ORs for the association between increased ZFAS1 expression and vascular invasion in cancer patients.

	High ZFAS1 expression		Low ZFAS1 expression			Odds Ratio	
Study or Subgroup	Events	Total	Events	Total	Weight	M-H, Fixed, 95% CI	Year
Wang W	50	79	38	80	35.8%	1.91 [1.01, 3.59]	2016
Fang C	15	36	6	37	8.9%	3.69 [1.23, 11.05]	2016
Nie F	15	27	11	27	12.6%	1.82 [0.62, 5.35]	2016
Tian FM	58	85	34	88	27.4%	3.41 [1.82, 6.38]	2016
Xia B	16	30	4	30	4.8%	7.43 [2.08, 26.55]	2017
Pan L	48	58	19	36	10.4%	4.29 [1.67, 11.05]	2017
**Total (95% CI)**		**315**		**298**	**100.0%**	**2.98 [2.12, 4.19]**	
Total events	202		112				

Heterogeneity: Chi² = 5.59, df = 5 (P = 0.35); I² = 11%
Test for overall effect: Z = 6.30 (P < 0.00001)

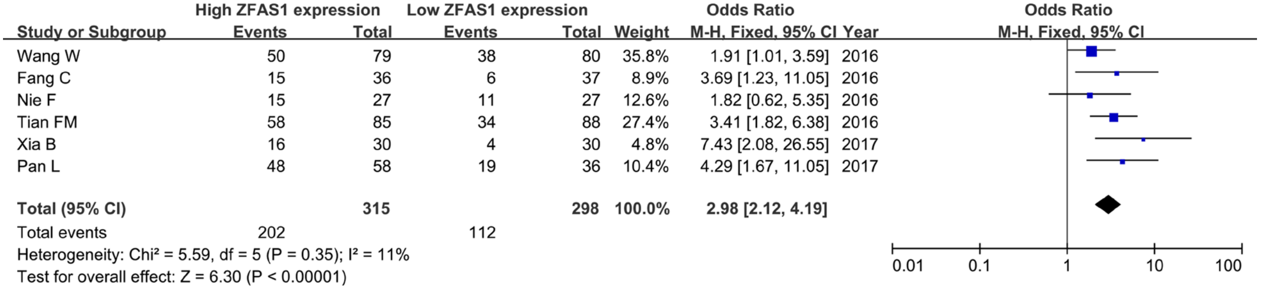

Supplementary Figure 2: Forest plot of ORs for the association between increased ZFAS1 expression and lymph node metastasis in cancer patients.

Study or Subgroup	High ZFAS1 expression Events	Total	Low ZFAS1 expression Events	Total	Weight	Odds Ratio M-H, Fixed, 95% CI	Year
Tian FM	51	85	30	88	26.9%	2.90 [1.56, 5.38]	2016
Wang W	50	79	38	80	31.7%	1.91 [1.01, 3.59]	2016
Fang C	17	36	8	37	9.5%	3.24 [1.17, 9.00]	2016
Nie F	22	27	8	27	3.4%	10.45 [2.92, 37.39]	2016
Pan L	43	56	19	35	12.4%	2.79 [1.12, 6.92]	2017
Xia B	22	30	12	30	7.3%	4.13 [1.39, 12.27]	2017
Lv QL	20	27	19	42	8.8%	3.46 [1.21, 9.92]	2017
**Total (95% CI)**		**340**		**339**	**100.0%**	**3.00 [2.18, 4.12]**	
Total events	225		134				

Heterogeneity: Chi² = 6.11, df = 6 (P = 0.41); I² = 2%
Test for overall effect: Z = 6.75 (P < 0.00001)

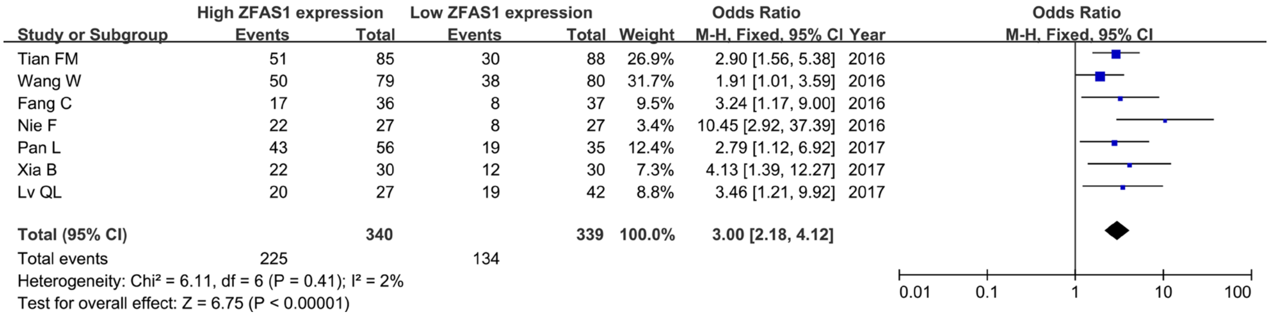

Supplementary Figure 3: Forest plot of ORs for the association between increased ZFAS1 expression and advanced TNM stage in cancer patients.

Study or Subgroup	High ZFAS1 expression Events	Total	Low ZFAS1 expression Events	Total	Weight	Odds Ratio M-H, Fixed, 95% CI	Year
Li T	40	57	35	56	12.5%	1.41 [0.64, 3.09]	2015
Fang C	14	36	22	37	15.8%	0.43 [0.17, 1.11]	2016
Nie F	15	27	11	27	5.8%	1.82 [0.62, 5.35]	2016
Tian FM	43	85	50	88	28.9%	0.78 [0.43, 1.42]	2016
Wang W	45	79	48	80	24.4%	0.88 [0.47, 1.66]	2016
Lv QL	17	27	21	42	7.2%	1.70 [0.63, 4.57]	2017
Pan L	29	37	17	23	5.4%	1.28 [0.38, 4.32]	2017
**Total (95% CI)**		**348**		**353**	**100.0%**	**0.98 [0.73, 1.33]**	
Total events	203		204				

Heterogeneity: Chi² = 7.05, df = 6 (P = 0.32); I² = 15%
Test for overall effect: Z = 0.11 (P = 0.91)

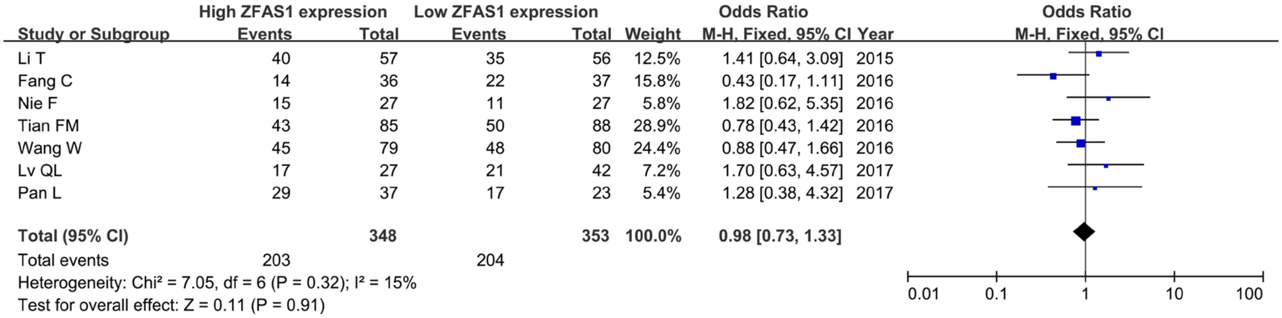

Supplementary Figure 4: Forest plot of ORs for the association between ZFAS1 expression and gender in cancer patients.

Study or Subgroup	High ZFAS1 expression Events	Total	Low ZFAS1 expression Events	Total	Weight	Odds Ratio M-H, Fixed, 95% CI	Year
Li T	29	57	25	56	22.4%	1.28 [0.61, 2.69]	2015
Nie F	19	27	10	27	5.4%	4.04 [1.30, 12.58]	2016
Tian FM	35	85	37	88	38.7%	0.96 [0.53, 1.77]	2016
Wang W	35	79	28	80	28.1%	1.48 [0.78, 2.80]	2016
Pan L	13	25	5	15	5.4%	2.17 [0.57, 8.19]	2017
**Total (95% CI)**		**273**		**266**	**100.0%**	**1.41 [1.00, 1.99]**	
Total events	131		105				

Heterogeneity: Chi² = 5.28, df = 4 (P = 0.26); I² = 24%
Test for overall effect: Z = 1.97 (P = 0.05)

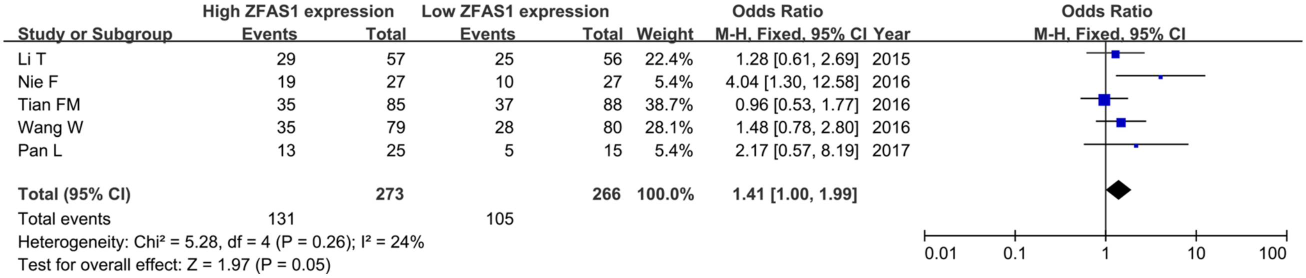

Supplementary Figure 5: Forest plot of ORs for the association between ZFAS1 expression and tumor size in cancer patients.

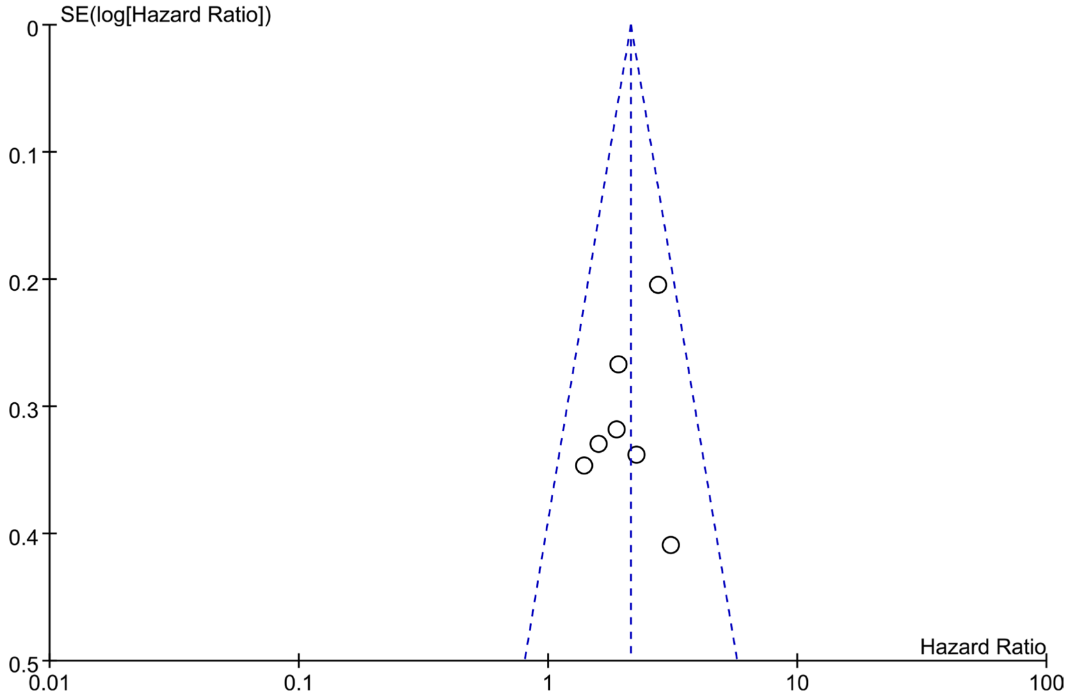

Supplementary Figure 6: Funnel plot of publication bias for the association between ZFAS1 expression and OS in cancer patients.

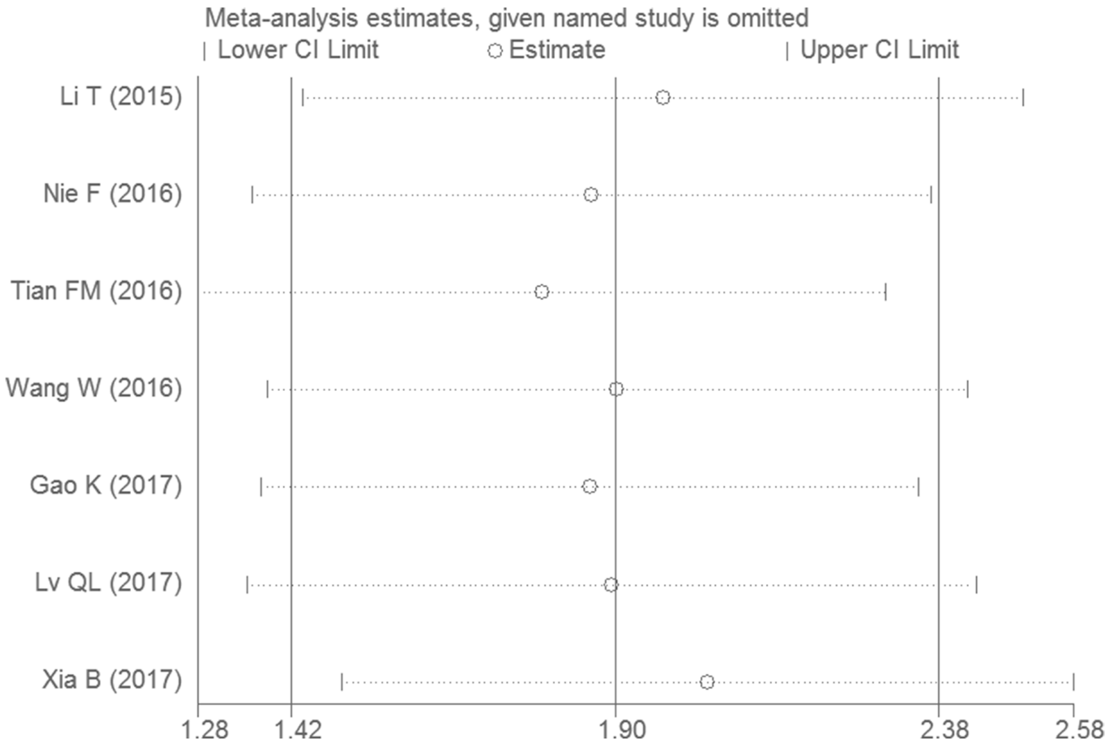

Supplementary Figure 7: Sensitivity analysis for the association between ZFAS1 expression and OS in cancer patients.
